# High-Throughput miRNA and mRNA Sequencing of Paired Colorectal Normal, Tumor and Metastasis Tissues and Bioinformatic Modeling of miRNA-1 Therapeutic Applications

**DOI:** 10.1371/journal.pone.0067461

**Published:** 2013-07-02

**Authors:** Christina Röhr, Martin Kerick, Axel Fischer, Alexander Kühn, Karl Kashofer, Bernd Timmermann, Andriani Daskalaki, Thomas Meinel, Dmitriy Drichel, Stefan T. Börno, Anja Nowka, Sylvia Krobitsch, Alice C. McHardy, Christina Kratsch, Tim Becker, Andrea Wunderlich, Christian Barmeyer, Christian Viertler, Kurt Zatloukal, Christoph Wierling, Hans Lehrach, Michal R. Schweiger

**Affiliations:** 1 Department of Vertebrate Genomics, Max Planck Institute for Molecular Genetics, Berlin, Germany; 2 Department of Biology, Chemistry and Pharmacy, Free University, Berlin, Germany; 3 Max Planck Institute for Molecular Plant Physiology, Potsdam-Golm, Germany; 4 Alacris Theranostics GmbH, Berlin, Germany; 5 Institute of Pathology, Medical University of Graz, Graz, Austria; 6 Next generation sequencing group, Max Planck Institute for Molecular Genetics, Berlin, Germany; 7 Structural Bioinformatics Group, Institute for Physiology, Charité–University Medicine Berlin, Berlin, Germany; 8 German Center for Neurodegenerative Diseases (DZNE), Bonn, Germany; 9 Otto Warburg Laboratories, Max Planck Institute for Molecular Genetics, Berlin, Germany; 10 Department of Algorithmic Bioinformatics, Heinrich-Heine University, Düsseldorf, Germany; 11 Institute for Medical Biometry, Informatics and Epidemiology, University of Bonn, Bonn, Germany; 12 Department of Gastroenterology, Charité–University Medicine Berlin, Berlin, Germany; Semmelweis University, Hungary

## Abstract

MiRNAs are discussed as diagnostic and therapeutic molecules. However, effective miRNA drug treatments with miRNAs are, so far, hampered by the complexity of the miRNA networks. To identify potential miRNA drugs in colorectal cancer, we profiled miRNA and mRNA expression in matching normal, tumor and metastasis tissues of eight patients by Illumina sequencing. We validated six miRNAs in a large tissue screen containing 16 additional tumor entities and identified miRNA-1, miRNA-129, miRNA-497 and miRNA-215 as constantly de-regulated within the majority of cancers. Of these, we investigated miRNA-1 as representative in a systems-biology simulation of cellular cancer models implemented in PyBioS and assessed the effects of depletion as well as overexpression in terms of miRNA-1 as a potential treatment option. In this system, miRNA-1 treatment reverted the disease phenotype with different effectiveness among the patients. Scoring the gene expression changes obtained through mRNA-Seq from the same patients we show that the combination of deep sequencing and systems biological modeling can help to identify patient-specific responses to miRNA treatments. We present this data as guideline for future pre-clinical assessments of new and personalized therapeutic options.

## Introduction

Colorectal cancer (CRC) is the third most common cancer worldwide and a major cause of cancer mortality with an incidence of approximately one million cases. At early stages a curative treatment is achieved by surgical resection and (neo-) adjuvant chemotherapy. Current chemotherapies however are often limited due to i) unspecific and broad mechanisms of action, ii) a large proportion of patients which is resistant to the chemotherapies, but nevertheless suffers from undesirable side-effects and iii) tumors that are diagnosed at advanced stages where a chemotherapy cannot be applied anymore.

Recent years have identified a number of oncogenic and tumor suppressive miRNAs [1]–[3]. MiRNAs are evolutionarily conserved, small (20–25 nucleotides) non-protein-coding molecules that regulate gene expression at the post-transcriptional level and participate in the regulation of various cellular processes, such as cell differentiation, cell cycle progression, metabolism and apoptosis [4]. The regulatory potential of miRNAs results from the target diversity of each miRNA, such that each miRNA targets multiple mRNAs and one mRNA can be regulated by different miRNAs.

Currently more than 1600 microRNA sequences have been identified in the human genome (miRBase v19, 2012) and it is predicted that ∼30% of the protein-encoding genes are regulated by miRNAs. In 2002 Calin et al. [1] showed that in over 65% of B cell chronic lymphocytic leukemia (B-CLL) patients, the miRNA genes miRNA-15 and miRNA-16 are deleted, suggestive of their tumor suppressor functions. Subsequently numerous miRNAs have been identified with oncogenic and tumor suppressive activities [2], [3].

For colorectal cancers miRNAs are not only grouped as tumor suppressors or oncogenes, but their specific functions in CRC-associated pathways has been identified [5]: As such, the adenomatous polyposis coli gene *APC* is targeted by the miR-135 family and results in a de-regulated Wnt/β-catenin pathway [6]. Other examples include *KRAS* which is targeted by the let-7 miRNA family, miRNA-18 and miRNA-143 which also targets *MACC1* (metastasis-associated in colon cancer-1). *MACC1* promotes tumor progression and metastasis through an activation of the HGF/Met signaling pathway [7]–[9]. MiRNA-21 is indicated as an oncogenic miRNA which can promote cell proliferation, inhibit apoptosis and enhance invasion and metastasis. Increased miRNA-21 expression has also been associated with inflammation in ulcerative colitis, thus linking inflammation and cancer [10], [11]. Interestingly, miRNAs can also function as kind of intermediate switch to transmit the effect of other proteins. TP53 for example directs the expression of miRNA-34 family members. TP53 induced miRNA-34a leads to apoptosis, cell cycle arrest and senescence [12], [13]. The upstream and downstream regulation of miRNAs adds another layer of complexity on the networks underlying CRC.

The complexity of biological systems favors computer models which have been developed with the aim to represent features of a disease and to predict therapeutic outcomes. One caveat of many such systems is that appropriate kinetic parameters need to be estimated. A Monte Carlo type strategy developed by Wierling and colleagues samples parameter vectors from a given random distribution with subsequent statistical significance testing [14]. Those mathematical models can integrate information of cellular processes, such as cellular signaling pathways, and can be used to study the qualitative and even quantitative behavior of the underlying biological system given specific perturbations, like targeted drugs or mutations. This approach can be used to study individual pathways, but also multiple signaling pathways taking into account cross-talk effects and the subsequent gene-regulatory network as well as regulatory effects of miRNAs [15].

We applied high throughput sequencing on colorectal normal, tumor and metastasis tissues to generate miRNA and gene expression patterns from individual patients. We identified miRNA-1, miRNA-129, miRNA-215, miRNA-497, miRNA-135b and miRNA-493 among our top candidates de-regulated in colorectal cancer. We extended our screen to 16 additional tumor entities (330 tissues) and found miRNA-1 constantly down-regulated. Using the Monte Carlo-based systems biology modeling approach we explored the power of miRNA-1 response prediction in individual patients and suggest a new strategy to optimize personalized treatment regimes.

## Materials and Methods

### Tissue Samples and Cell Lines

The primary colon carcinoma tissue and matched normal colon epithelium as well as liver metastases tissue used for the next generation sequencing (NGS) experiments were obtained from patients diagnosed with CRC and undergoing surgical resection (**Table 1** **, Table S1, Methods S1**). The study has been approved by the Research Ethics Committee of the Medical University of Graz. Colon cancer cell lines SW480 and SW620 were obtained from American Type Culture Collection (ATCC). All cells were maintained and propagated according to the recommendations of ATCC.

**Table 1:** Clinical parameters of the colorectal cancer patients.

Patient	Sex	Age	MS^a^-Status	Tissue Type	Organ
P1	M	70	stable	Tumor	Sigmoid
				Metastases	Liver
P2	M	74	stable	Tumor	Coecum
				Metastases	Liver
P3	M	44	instable	Tumor	Colon asc.
				Metastases	Liver
P4	W	49	stable	Tumor	Rectum/Sigmoid
				Metastases	Liver
P5	W	80	instable	Tumor	Colon
				Metastases	Lymph node
P6	W	66	stable	Tumor	Colon asc.
				Metastases	Liver
P7	W	76	stable	Tumor	Colon asc.
				Metastases	Lymph node
P8	M	74	stable	Tumor	Coecum
				Metastases	Liver

a microsatellite.

For the large cancer screen 243 tumor samples and 87 normal tissue samples were selected from the biobank of the Medical University of Graz, Austria. This collection consists of tumor and normal tissue samples obtained from different organs, such as adipose tissue, brain, breast, colon, endometrium, kidney, liver, lung, lymphatic system, muscle, ovary, pancreas, prostate, stomach, testis and thyroid gland (**Table S2**). All tissue samples were reviewed and evaluated histopathologically and a macrodissections of the required areas were performed before RNA extraction.

### RNA Isolation from Human Cell Lines

RNA was extracted with the Trizol (Invitrogen) reagent according to the manufacture’s protocol including a DNase digestion step with the RNase-free DNase set from Qiagen (**Methods S1**). The integrity was assessed using the Agilent BioAnalyzer 2100 technology.

### Library Preparation and Next Generation Sequencing of RNAs and Small RNAs

For RNA sequencing rRNA substraction was performed with the RiboMinus™ Human/Mouse Transcriptome Isolation Kit SmallRNA and RNA sequencing including smallRNA isolation, cDNA library preparation, and sequencing was performed by Illumina’s DGE smallRNA sample and Illumina’s RNA-Seq prep kit following the manufacturer’s instructions (see also **Methods S1**). The purified DNA was quantified and diluted to 10nM for cluster generation and sequencing on an Illumina Genome Analyzer GAII. The data discussed in this publication have been deposited in NCBI’s Gene Expression Omnibus and are accessible through GEO Series accession number GSE46622 [16].

### Analysis of Deep Sequencing Data

Primary data analysis: Sequences were obtained using Illumina Genome Analyser IIx. Reads were mapped against Illumina adaptor sequences using blat [17] and adaptor signatures were clipped from the reads subsequently. In detail; clipping always starts from one of the ends of a read and reads with a length less than 16 bp after clipping were omitted from analysis.

Analysis of miRNA expression: Adaptor-clipped reads were mapped to the human genome version GRCh37 (hg19) with the bwa 0.5.8 alignment tool [18] using default parameters. As a measure for miRNA expression, reads on target regions were counted. A read had to have at least one base within the target region to be evaluated “on target”. Log2 ratios were calculated using the read counts per target to compare different conditions. Ratios were subsequently normalized for each comparison by centering the median of log2 ratios to zero. Differential expression was evaluated using student's t-test on the log2 ratios (samples versus controls).

Evaluation of miRNA biomarker combinations: To assess the potentials of combinations of miRNAs for the discrimination of sample subgroups Mann-Whitney p-values were calculated for each miRNA comparing tumor and normal, metastasis and normal, tumor and metastasis, and tumor/metastasis and normal. For each comparison, the 50 best scoring miRNAs were extracted. Linear combinations of each two miRNAs out of the 50 were assessed for separating the groups. If no overlap between the groups was detected using a marker combination, this marker combination was called “promising”.

Analysis of mRNA expression: Adaptor-clipped reads were mapped to the human genome version GRCh37 (hg19) using transcript models taken from Ensembl v64 with TopHat [19]. Using the parameters -u -N –max-bundle-length 350000000–max-bundle-frags 50000000. Differential expression as well as unknown transcript models were determined using the Cufflinks software bundle [20]. In detail transcript models from all tissues were merged for each patient using cuffcompare and differential expression was determined using cuffdiff with upper quartile normalization.

### Real-time Quantification of microRNAs using Stem-loop real-time PCR

For real-time quantification of mature microRNAs we used TaqMan® MicroRNA Assays (Applied Biosystems) and performed a two-step RT-PCR according to the manufacturer’s protocol (see also **Methods S1**). All reactions were typically run in triplicates. The relative quantification of expression was calculated by the "delta CT" method using RNU44 as an internal control [21]. (For an assessment of RNU44 as reference gene see **Methods S1**.) The comparison of normal and tumor or metastasis tissues was calculated as “delta delta Ct” values.

### Transfection of Cells with miRNA-1 Mimic and Functional Analyses

Transfection of SW480 and SW620 cells was performed with 100nM microRNA-1 mimic (Dharmacon) using Hiperfect transfection reagent (Qiagen). For wound healing experiments, SW480 and SW620 cells were seeded, after 24 h transfected and additional 24 h later wounds were generated through the monolayer with a pipette tip. Wound closure was assessed after 24 h and 48 h. For cell viability assays SW480 and SW620 cells were seeded into 96 well plates at concentrations of 5000 cells/well and transfected with the corresponding miRNA mimic. For Camptothecin treatment cells were additionally treated for 24 h and 48 h with 0.06 µM Camptothecin. For cell viability the alamarBlue reagent was used according to the manufacturer’s protocol.

### Model Description

The development of the mathematical model of cellular signaling pathways was guided by the cancer-related pathways as described for the hallmarks of cancer by Hanahan and Weinberg [22], [23]. Ligands and receptors as well as downstream signaling pathways that have been integrated are listed in **Table S3** and the complete model is provided as an SBML file (**Data S1**). By rigorous literature screening using different resources, like PubMed, GeneCards, iHOP and Bibliosphere, molecular interaction details were added when required. The model integrates several receptor tyrosine kinases such as epidermal growth factor receptor (EGFR), insulin-like growth factor receptor (IGF), colony stimulating factor receptor (CSFR), platelet-derived growth factor (PDGF), vascular endothelial growth factor (VEGF), insulin receptor (INSR) and their respective ligands as well as their subsequent signal transduction via the MAP kinase cascade and PI3K/AKT. Furthermore, the model covers signaling that is triggered by the ligands transforming growth factor-β (TGFb1), bone morphogenetic protein (BMP) and its corresponding signaling via SMADs, by different interleukins, by WNT and its subsequent signal transduction via APC/AXIN/GSK3β/β-catenin, by interferone via JAK/STAT, and by Delta/Notch and the corresponding signal transduction via the notch intracellular domain. Moreover, the model contains several direct transcriptional targets of the individual signal transduction pathways as described in text books or as annotated by Transfac [24]. The model also covers signaling that triggers apoptosis via the ligands FASL, TNF**α**, and TRAIL. The model integrates in total 3542 reactions and 2369 components covering 505 human genes. The respective ODE model has 3845 kinetic parameters, 1737 variables and 632 components that are treated as fixed. The Monte Carlo-based simulations of the model were performed as described in Wierling et al. [14].

## Results

### High Throughput Small RNA Sequencing of Matched Normal, Tumor and Metastasis Colon Tissues

To identify miRNAs which are constantly over- or under-expressed in tumor- and metastasis tissues we screened matching normal, tumor and metastasis tissues from eight colorectal cancer patients (**Table 1** **, Table S1**). We generated genome-wide miRNA expression maps with the Illumina high throughput sequencing technology. For miRNA expression analysis we aimed to sequence more than 25 million reads total for each sample of which 71.34% of uniquely aligned reads are located on miRNA regions as taken from miRBase release 18 (**Table S4**). We detected 724 miRNAs covered with at least one sequencing read in at least one tissue or cell line over all experiments. Our high-throughput sequencing approach proved to be very reliable as was probed using technical replicates and RT-PCR data (**Data S2**).

**Figure 1:**
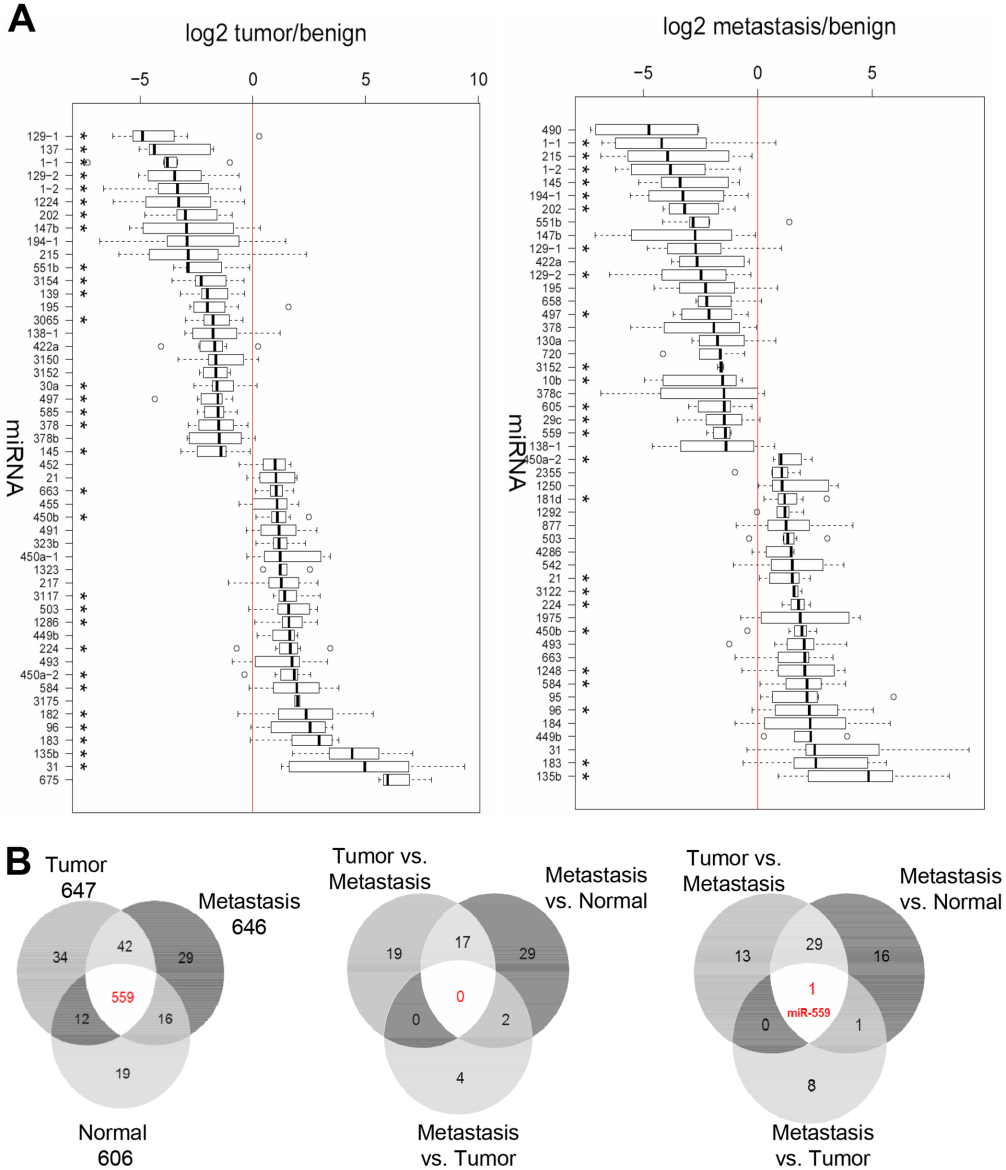
Differential expression of miRNAs in colon tumor and metastasis tissues. (**A**) Top 25 up- and down-regulated miRNAs comparing tumor (left) or metastasis (right) tissues versus normal colon samples as analyzed by Illumina sequencing. All depicted miRNAs sufficed a p-value threshold ≤0.05. A star indicates samples with p≤0.01. (**B**) Venn diagram of microRNAs expressed in colorectal cancer patients, as determined by Illumina sequencing. (Left) Numbers of detected miRNAs, specific for each tissue (normal (N) = 19, tumor (T) = 34, metastases (M) = 29) and in all tissues (559). (Middle) Venn diagram of the significantly up-regulated miRNAs (p-value ≤0.05) for all comparisons (N/T, N/M and T/M). (Right) Venn diagram of the significantly down-regulated miRNAs (p-value ≤0.05) for all comparisons (N/T, N/M and T/M).

We identified several miRNAs to be differentially regulated in tumor and metastasis tissues, some of which have already been described in the literature, which further supports the validity of our approach. We found a 21-fold up-regulation of miRNA-135b in the colon cancer samples (p-value = 0.0001) which has been described [6] and a 28-fold higher expression in metastasis tissues (FC = 28.94, p-value = 0.00156) (**Figure 1A** **, Table S5, S6**). Other findings which concord with the literature are the down-regulation of miRNA-145 and miRNA-133b and the up-regulation of miRNA-21, miRNA-31, miRNA-183 and miRNA-96 [25], [26]. When compared to the normal tissues we found 19 miRNAs specifically up-regulated and 13 down-regulated in the tumor tissues and 29 miRNAs specifically up-regulated and 16 down-regulated in the metastasis tissues. We identified one microRNA, miRNA-559, which is down-regulated in all tumor tissues with an even stronger and significant down-regulation in the metastasis. MiRNA-559 targets the protooncogene human epidermal growth factor receptor 2 (*HER2*) which leads to cell growth and differentiation [27].

Primary tumor and corresponding metastasis were found to differ less from each other than in comparison to the normal tissue samples, a finding which is also supported by hierarchical clustering analyses (**Figure S1A**). We identified only six miRNAs up- and 11 down-regulated in the metastasis as compared to the corresponding tumor tissue (**Figure S1B, Table S5C, S6**).

### MiRNA-1 is Constantly Down-regulated in 16 Different Tumor Entities and Acts as Tumor Suppressor

Potential miRNA drug candidates should be de-regulated in both tumor and metastasis tissues. As top candidates we determined miRNA-1, miRNA-129, miRNA-135b, miRNA-215, miRNA-493 and miRNA-497. We screened these six candidates in 16 additional tumor entities using 330 different tissue samples. Normal and tumor tissues were taken from adipose tissue, brain, breast, colon, endometrium, kidney, liver, lung, lymph node, muscle, ovary, pancreas, prostate, stomach, testis and thyroid gland (Table S2).

**Figure 2:**
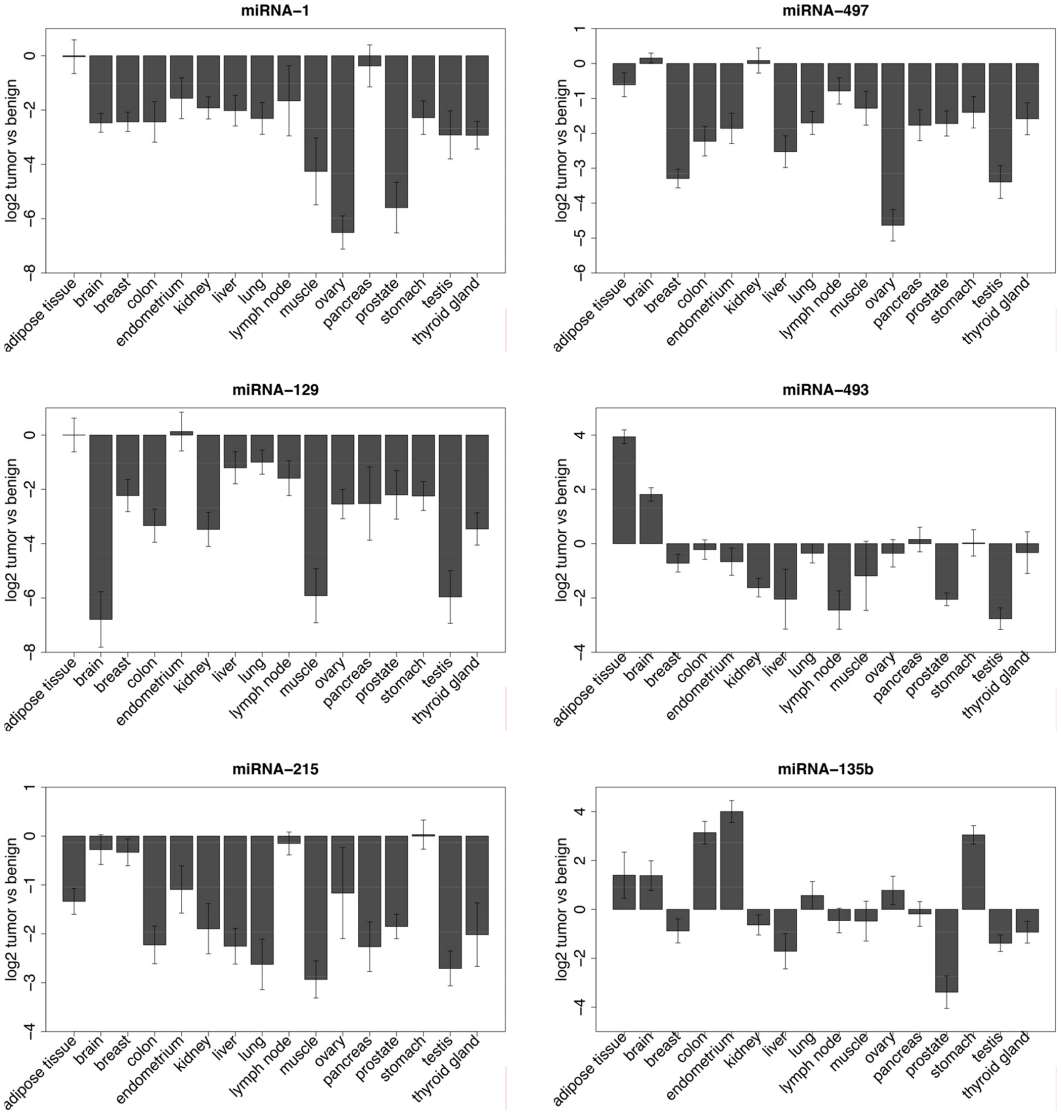
Expression of miRNA-1, miRNA-129, miRNA-215, miRNA-135b, miRNA-493 and miRNA-497 in 16 different cancer entities using the TaqMan platform. Expression values of both miRNAs were determined with the delta delta Ct method. Normalizations were performed against a stable internal control gene (RNU44) and to the expression levels in the normal tissues.

**Figure 3:**
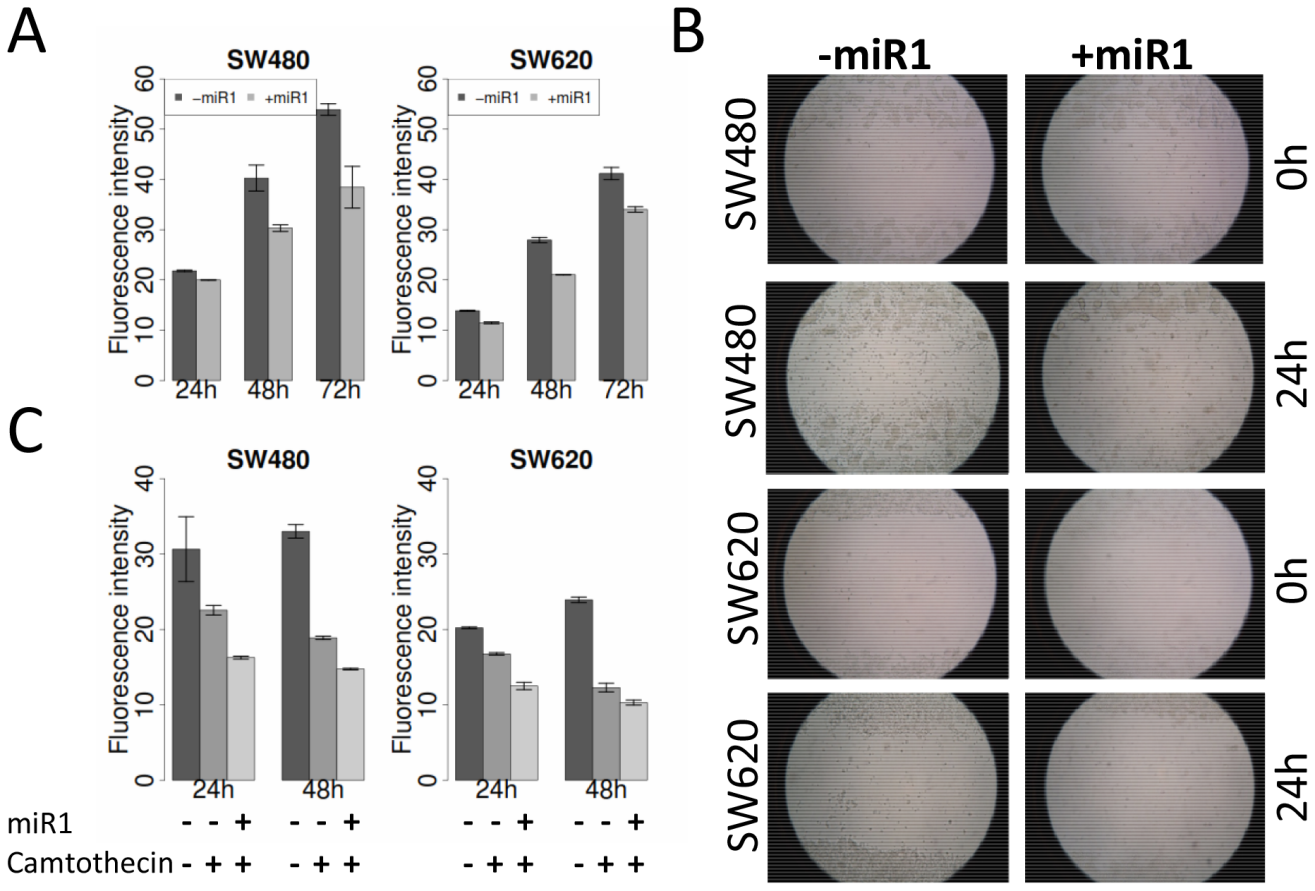
Functional assays on miRNA-1 as a potential tumor-suppressor gene. (**A**) AlamarBlue cell viability assay to test the effect of miRNA-1. SW480 (primary colon cancer cell line) and SW620 (corresponding metastases cell line) cells were transfected with miRNA-1 mimics (+miR-1) or mock transfected (−miR-1) and measured using an spectrophotometer after 24 h, 48 h and 72 h. The miRNA-1 level was determined by TaqMan assays for mature microRNAs. (**B**) “Wound healing” assay for miRNA-1 in SW480 and SW620 cells. After 24 h of transfection with miRNA-1 mimics a uniform scratch was generated through each confluent cell layer and “wound” closure was documented after 24 h using a phase-contrast microscope (n = 2). (**C**) AlamarBlue cell viability assay in SW480 and SW620 cells after camptothecin treatment alone or in combination with miRNA-1. Cell viability was measured after 0 h, 24 h and 48 h of drug treatment using a spectrophotometer.

We found miRNA-1, miRNA-129, miRNA-215 and miRNA-497 down-regulated over the majority of cancers. The most significant down-regulations were found for miRNA-1 in colorectal cancer (fold change = 8.5), muscle (fold change = 20.28), ovary (fold change = 88.87) and prostate tissues (fold change = 45.94). The expression of miRNA-135b and miRNA-493 varied across all cancer tissues investigated. For miRNA-135b we observed in colon cancer an up-regulation (fold change = 9.23), similar in endometrium (fold change = 15.44), stomach (fold change = 5.7), adipose tissue, brain, lung and ovary (**Figure 2**). In contrast, breast, kidney, liver, lymph node, muscle, pancreas, prostate, testis and thyroid gland tissues showed down-regulation of miRNA-135b expression in tumors. Based on the constant de-regulation across many tissues we concentrated on miRNA-1 for further experiments. Ectopic expression of miRNA-1 in lung, liver and colorectal cancer as well as in rhabdomyosarcoma inhibits cellular growth similar to the function of a classical tumor suppressor gene [28]–[32]. To investigate the functional impact of miRNA-1 in a tumor-metastasis model system we used the colorectal cancer cell lines SW480 (primary tumor) and SW620 (metastasis) which originate from the same patient and thus best resemble our normal-tumor-metastasis sequence. We found miRNA-1 down-regulated in SW480 (fold-change = 61) and SW620 cells (fold-change = 7) when compared to the averaged expression of all normal colon tissues. We transfected these cell lines with miRNA-1 mimics to increase the miRNA-1 level and monitored the cell viability with an alamarBlue assay. Measuring the fluorescence intensity of metabolizing cells we detected a decrease of approximately 25% after 48 hours and of 30% after 72 hours for SW480 and of 20% after 72 hours for SW620 cells after miRNA-1 transfection (**Figure 3A**). We also examined the motility of miRNA-1 transfected colon cancer cells (SW480, SW620) using a “scratch wound healing” assay and found that miRNA-1 expressing cells migrated towards the “wound” at a much slower rate (**Figure 3B**).

A major problem in oncology is the therapy-resistance of many tumors: Recent studies showed that miRNAs can alter the sensitivity of cancer cells to therapeutic agents [33]–[38]. MiRNA-1 has been found to act synergistically with doxorubicin in lung cancer cells [29]. We were interested if miRNA-1 can also alter the sensitivity of colorectal cancer cells to anticancer drugs. We treated SW480 and SW620 cell lines with camptothecin, a topoisomerase inhibitor, in presence and absence of miRNA-1 and measured the cell viability with an alamarBlue assay after 24 h and 48 h of treatment. Compared to the non-transfected cells we detected a 8% and 17% reduced viability after 24 hours camptothecin treatment in SW480 and SW620 cells, respectively (**Figure 3C**). Combination of camptothecin treatment and expression of miRNA-1 further lowered the cancer cell viability by 72% and 75%, respectively. The effect was even stronger after 48 hours: Camptothecin treatment alone reduced the cell viability to 57% and 51% in SW480 and SW620, respectively. A combination therapy with miRNA-1 further decreased the viability by 45% and 43%. Thus, in both cell lines camptothecin and miRNA-1 had an additive effect.

### Modeling of miRNA-1 Function and Therapeutic Effects

We showed that miRNA-1 is down-regulated in many different tumor entities and exerts tumor suppressor like characteristics. Consequently, augmenting the expression level of miRNA-1 might revert tumor cells.

To investigate the effect of miRNA-1 expression on a system biological scale we used a computer simulation of cancer related cellular signaling pathways [22], [23] and its down-stream regulated genes. The mathematical model was developed with PyBioS that is a web-based software for the modeling and simulation of cellular reaction systems [14], [39], [40]. It provides a framework for the development of computational models using an interface to public pathway databases such as KEGG, Reactome and ConsensusPathDB [41]–[43]. PyBioS supports the automatic generation of ordinary differential equations (ODEs) system of the model using a given set of kinetic laws and their respective kinetic parameters. The ODE system of the model can subsequently be used for simulation by numerical integration and model analysis. The model comprises more than 4,000 components representing functional interactions of approximately 500 genes.

**Figure 4:**
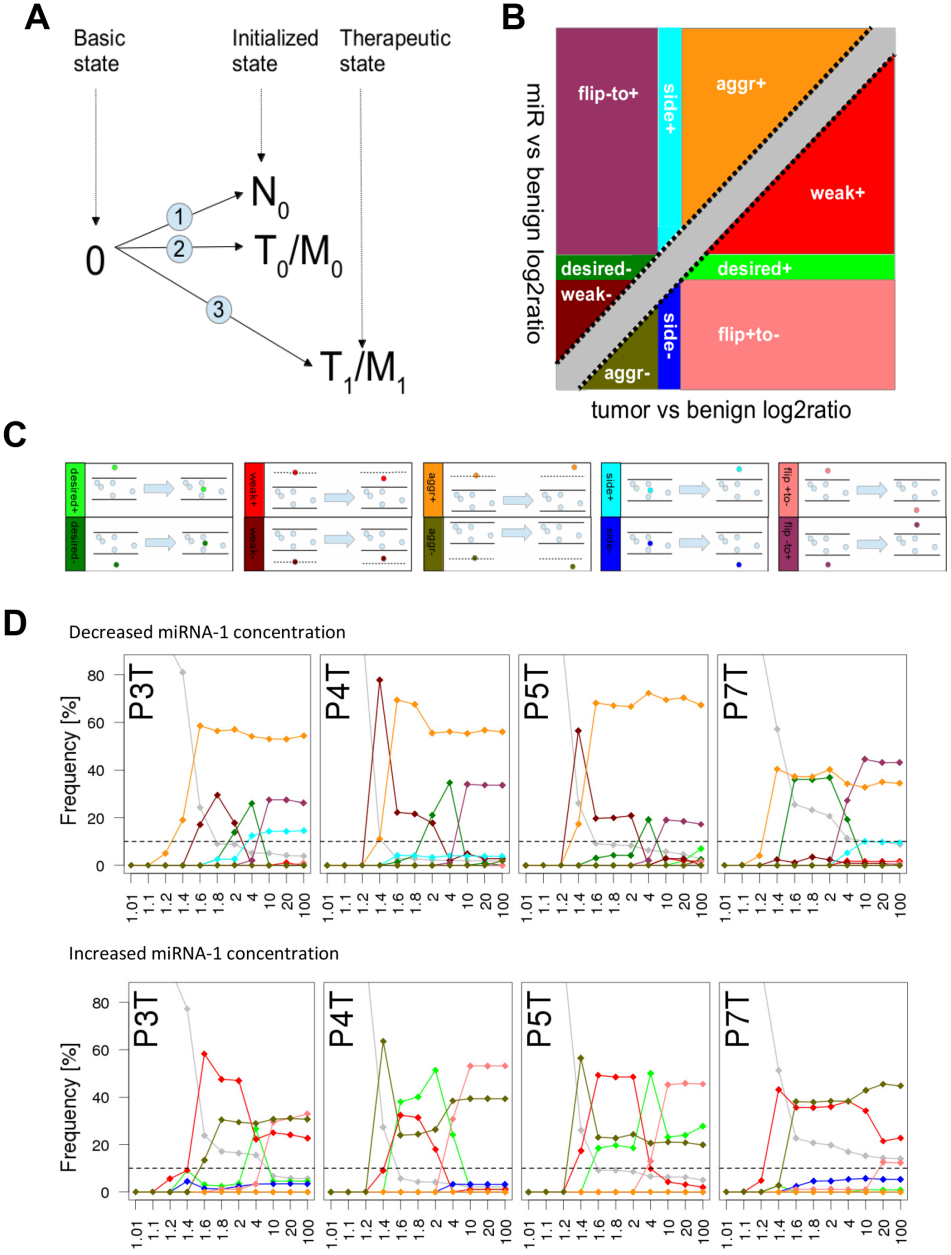
*In silico* modeling of the individual response of 4 patients to either miRNA-1 down-regulation or miRNA-1 drug treatment using a Monte Carlo-based computational cancer model integrated in PyBios. (**A**) Schematics of the modeling approach: mRNA-Seq data from the tumor or metastasis of each patient was used to initialize the *in silico* model (T_0_/M_0_). After ‘treatment’ of the model with different miRNA concentrations, the ‘therapeutic state’ model (T_1_/M_1_) was compared to the mRNA-Seq data of the normal tissue of the same patient (N_0_). (**B,C**) We compared the expression changes induced by miRNA-1 dosages to the expression changes originally found in the tumor. In both cases changes were calculated in comparison to the normal tissue expression as log2ratios (T-N and miR-N). Predicted component concentration changes in the model were classified into five different groups (or 10 different sub-groups) depending on the changes between the T-N and miR-N log2ratios: **‘**Desired’ (desired therapeutic effect, component concentration levels back to 'normal'), ‘weak effect’ (weak effect in changing the component concentration but tendency to 'normal'), ‘side effect’ (negative therapeutic effect), ‘aggravated’ (component concentration aggravated in the same direction) and ‘flip’ (component concentration status flips from up- to down-regulated or vice versa). Each group can be divided into two sub-groups ‘plus’ and ‘minus’ which depicts an initial up- or down-regulation of a component in the tumor normal comparison as determined by a log2ratio cutoff greater than 0.58 or smaller than −0.58 respectively. (**D**) miRNA-1 was either decreased (top row) or increased (bottom row) for the tumor tissues of patient 3, 4, 5, and 7 and gene expression changes were observed in a dose-dependent manner. Different concentrations of miRNA-1 are visualized as scatter plots where the frequency reflects the ratio of the number of compounds contained in each classification-subgroup to the sum of all compounds; only those components were considered which were either higher than 1.2-fold up-regulated or lower than 0.8-fold down-regulated in comparison to the control state.

We modeled the effect of miRNA-1 up- and down-regulation for four patients. For each of the patients we had generated mRNA-Seq data for each tissue, normal, tumor and metastasis from the same samples used for the miRNA analysis. The RNA expression levels were used to initialize the gene expression state of the model states normal N_0_, tumor T_0_ and metastasis M_0_ for each patient individually (**Figure 4A** **, Table S7**). In order to investigate the effect of miRNA-1 overexpression and repression on the biological system of an individual, we screened our cancer model for target genes of miRNA-1. According to the miRWalk database (version March 15^th^ 2011, [44]) 843 genes are known as validated targets of miRNA-1, of which 59 are integrated in our cancer model (**Table S8**). To study the quantitative effect of different miRNA-1 levels, we simulated different levels of the miRNA target genes and extracted the expression levels of all 1645 components contained within the model (**Table S9, S10**). Outputs of the simulations are steady state levels of signaling components and mRNA values of their direct downstream regulated genes which we used to calculate the effect of different miRNA-1 levels in terms of changed expression. The changed expression levels for all components in the model including downstream regulated mRNAs and excluding direct miRNA-1 targets were then evaluated according to whether the effect was desired or adverse. In detail, differential expression was calculated as comparison between tumor (T_0_) and normal state (N_0_) as well as between treated (T_1_ to T_y_ or T_1_ to T_z_) and normal (N_0_) states (**Figure 4A**). The alterations were stratified into five different groups: ‘desired’, ‘aggravating’, ‘side effect’ and ‘weak effect’, each of which was subdivided by the direction of change (‘plus’ and ‘minus’) (**Figure 4B, C**). The alteration ‘flip’ was assigned if the direction of change ‘plus to minus’ or ‘minus to plus’ was observed.

Genes in the tumor that approached the normal state were summed up as ‘desired’. Genes with a more severe expression level after treatment were counted as ‘aggravated’. If the expression levels of genes were the same in tumor and normal tissue and after treatment changed to an up- or down-regulation, the genes were summarized as ‘side effect’. The numbers of genes in five effect-groups were calculated for each manipulated miRNA-1 concentration and dosage-dependent curves were plotted (**Figure 4D** **, Table S11**). First we simulated a down-regulation of miRNA-1 (1.01 to 100fold) mimicking the disease state. For the majority of genes we found an amplification of their over-expressed state (‘aggravated plus’). Besides the pronounced tumor-like effect of a low miRNA-1 level, many genes were also counted as ‘desired minus’ and ‘weak minus’, indicating that gene expression levels of previously de-regulated states returned towards the normal state after miRNA-1 treatment **(** **Figure 4D** **, Figure S2)**.

To analyze the potential therapeutic effect of miRNA-1 overexpression we simulated an increase in miRNA-1 levels by down-regulating the synthesis rate of miRNA-1 targets from 1.01- to 100-fold. In particular patients 4 and 5 had many genes with a desired expression change which were grouped into ‘weak effect plus’ and ‘desired plus’. The optimal therapeutic window was found between 1.6 and approximately 4-fold change of miRNA-1 levels. Interestingly, at 1.2 to 1.5 fold down-regulation of miRNA-1 the fraction of genes with an enhancement of their decreased expression (‘aggravated minus’) predominated. The weakest positive effect of miRNA-1 down-regulation was detectable for patient 7 (low ‘weak+’ and no ‘desired+’), indicating that a potential miRNA-1 treatment might not be beneficial for this patient.

## Discussion

In recent years miRNAs have emerged as diagnostic and prognostic biomarkers, but also as potential new therapeutic molecules. In this study on colorectal cancer we investigated the complete set of miRNAs by means of high throughput sequencing technology in normal, tumor and metastasis tissue samples originating from the same patients. The inclusion of tumor and metastasis tissues from the same patient represents a unique resource not investigated with high throughput technologies so far. Many studies have analyzed miRNA profiles in colorectal cancer, but either applied real-time PCR or array-based technologies, or were restricted to normal/tumor pairs [25], [45]–[48]. Nevertheless we found a broad overlap between already published miRNA regulations and our data. For the comparison we used the PhenomiR 2.0. PhenomiR is a database which provides information about differentially deregulated miRNAs and is generated by manual extraction of data out of more than 365 scientific articles [49]. We found that out of 64 which have been described in PhenomiR to be overexpressed 43 were also overexpressed in our experimental data. On the other side, out of 49 down-regulated miRNAs in PhenomiR we found 35 also down-regulated in our data. If we also apply a p-value restriction (p-value <0.05) we have 79 miRNAs left in our analysis. Of these 30 have an entry (down-regulated, overexpressed or ambiguous) in the database. PhenomiR lists 10 of them as overexpressed and out of these we could validate 7 with our sequencing study. As down-regulated miRNAs PhenomiR lists 12 miRNAs out of which 10 can be confirmed by our approach. Among the top candidates are miR-135b, miRNA-143 and miRNA-21 (Table S12).

The small RNA sequencing data revealed a total of 29 miRNAs significantly down-regulated and of 17 miRNAs up-regulated in tumor and metastases as compared to the normal tissues. Overall, we found more differentially expressed miRNAs in metastases than in primary tumors, indicating that metastases undergo further genomic alterations that lead to changed miRNA patterns. For a potential use as biomarker candidates we were interested to see if combinations of two miRNAs would be sufficient to discriminate normal and malign tissues. In this line, among the top 50 differentially expressed miRNAs we found 143 (T/N) and 159 (M/N) different combinations of two miRNAs which could serve as potential biomarkers to separate normal from primary tumor or metastases tissues (**Figure S3**).

One of the aims of our study was to identify 'driver miRNAs' and we reasoned that miRNAs which are constantly de-regulated in different tumor types might have a central carcinogenic role. We performed a large tissue screen with 16 different tumor types for miRNA-1 miRNA-135, miRNA-129, miRNA-215, miRNA-493 and miRNA-497. In general we found a constant decrease in all tumor tissues for down-regulated miRNAs (miRNA-1, miRNA-129, miRNA-215 and miRNA-497) (Figure 2 and data not shown), whereas for miRNA-135b and miRNA-493 the patterns are more heterogeneous between the tumor tissues. A down-regulation of microRNAs has been described as a common phenomenon and thus might play a causal role in the generation or maintenance of tumors [50]. The impaired miRNA expression is most likely due to an altered miRNA processing machinery which also promotes oncogenic transformation [51].

For further analyses we selected miRNA-1 as top drug candidate based on the findings that miRNA-1 i) is strongly down-regulated in colorectal tumors and metastases, ii) is constantly down-regulated over 16 different tumor entities and iii) acts as tumor suppressor in functional assays and iv) exerts camptothecin-additive functions. MiRNA-1 is known to be expressed in cardiac and skeletal muscle cells and inhibits cell cycle progression by interacting with the histone deacetylase 4 (HDAC4) and the serum response factor (SRF) [52], [53]. In rhabdomyosarcoma miRNA-1 suppresses tumor growth by targeting the oncogene c-met [30] and in A549 lung cancer cells an over-expression of miRNA-1 sensitizes cells to the chemotherapeutic agent doxorubicin [29]. Here miRNA-1 induces apoptosis by an enhanced activation of caspases 3 and 7 and depletion of anti-apoptotic Mcl-1. Assuming that miRNA-1 could also have a triggering effect on colon cancer we applied *in silico* analysis to address the question.

We used a systems biology approach, implemented in the PyBioS modeling software to emulate the signaling network influenced by miRNA-1. We simulated the effect of a wide range of miRNA-1 concentrations: decreased concentrations to mimic the state of tumors and increased concentrations to assess the potential therapeutic effects of miRNA-1. Before we changed the miRNA-concentrations we initialized our computer model with mRNA-Seq data generated from the tumor colonic tissue simulating an *in silico* tumor. We then modulated the miRNA-concentrations in the model and measured *in silico* gene expressions. These we compared with the mRNA-Seq data generated from the normal colonic mucosa from the same tumor patient and evaluated if the gene expression shift was towards a normalization or indicated an adverse effect (Figure 4A).

The possible effects of altered miRNA-1 levels on gene expression were grouped into five classes (ten subclasses) and we counted how many genes were predicted to belong to each class (Figure 4B,C). As expected, a simulated down-regulation of miRNA-1 (1.01- to 100-fold) led to a significant number of up-regulated genes. Interestingly, the up-regulations were even more pronounced in the simulation indicating a continuation of the carcinogenic process (‘aggr.plus’, orange in Figure 4B, C).

The simulation of miRNA-1 as a therapeutic agent was accomplished by gradually decreasing protein synthesis rates of miRNA-1 target genes. The group of genes with the desired effect (‘desired +’) - tumor expression reduced to 'normal' levels - showed a dose dependent reaction to increasing miRNA-1 concentrations. A similar pattern was found for genes where the effect was too weak to revert tumor expression levels back to 'normal', but nevertheless, the direction of change was desirable (‘weak +’). On the other side, we also found genes with a significant decrease of their expression below the ‘normal’ state in a sense of ‘overtreatment’ (‘flip+to−‘). Another set of genes was already down-regulated in the tumor and was even more down-regulated after increased miRNA levels which represents potentially undesired side effects (‘aggr−‘). This fraction is disproportionately high in patient 4 and 5 at miRNA concentrations of 1.2 to 1.6fold increase and might indicate that a miRNA-1 treatment at this concentration would not be beneficial for these patients and that there is an optimal therapeutic window between 1.6 and 4fold increase of miRNA-1. For patient 3 and even more for patient 7 complete beneficial effects (‘desired+’) are low, thus these patients might respond less to a miRNA-1 increase. Interestingly, in our miRNA-Seq approach we find miRNA-1 on average 10 to 13 fold downregulated. For the *in silico* modeling similar concentrations of 1.2 to 4 fold upregulation are effective. The slight discrepancy between the values may be explained by the different systems investigated – a biological system and a semi-quantitative computer-based prediction using a Monte Carlo based approach.

Since we have used patient - individual RNA expression levels for the initialization of each tissue within the model we were able to investigate the effect of a change in miRNA-1 individually. Although in its infancy, this approach could be scaled up for a large number of patients. In contrast, *in vivo* investigations of functional miRNA effects are not feasible; in particular not for each patient separately as it would be required in the clinic. These experiments would take too long, would be too expensive and too work-intense for routine clinical use.

The predicted therapeutic effects we described in this study are already promising given the fact that we have only used one miRNA as therapeutic candidate. However, we suppose that a combination of several miRNAs can potentiate the positive effect, not only because each miRNA might be applied in concentrations closer to the 'normal', but also because therapeutic results might have a synergistic effect.

Taken together, we used mRNA-Seq data for each tissue - normal, tumor and metastasis – of each patient individually and predicted the response of miRNA-1 substitution *in silico.* By using combined miRNA- and mRNA-Seq data from the same tissue we were able to monitor not only patient-specific, but also to take into account tissue-specific effects. The results of our systems biology approach have shown a patient-specific response to miRNA treatment. We propose that this *in silico* modeling approach in combination with mRNA−/miRNA- Seq data is an effective strategy for the design of an individualized cancer treatment – both for the selection of the right treatment as well as the identification of the optimal therapeutic range.

## Supporting Information

Figure S1 **Changes in miRNA expression between normal, tumor and metastasis tissues.**
(PPT)

Figure S2 **Modeling of the miRNA down- and upregulation in metastasis tissues of patient 3,4,5 and 7.**
(PPT)

Figure S3 **Marker combinations for the discrimination of normal/tumor, normal/metastasis, normal/tumor and metastasis and tumor/metastasis tissues.**
(PPT)

Table S1 **Complete clinical information of the colorectal cancer patients.**
(XLS)

Table S2 **Clinical information of samples for the screen of different tumor entities.**
(XLS)

Table S3 **Ligands and receptors as well as downstream signaling pathways integrated in the computer model.**
(XLS)

Table S4 **Small RNA sequencing statistic.**
(XLS)

Table S5 **List of 25 most significantly differentially expressed miRNAs N/T, N/M, T/M.**
(XLS)

Table S6 **Complete lists of miRNAs detected in (A) N/T, (B) N/M and (C) T/M.**
(XLS)

Table S7 **mRNA sequencing statistic for colon cancer patients.**
(XLS)

Table S8 **Targets of miRNA-1.**
(XLS)

Table S9 **The initial conditions of miRNA-1 down-regulation of an individual sample.**
(XLS)

Table S10 **The initial conditions of miRNA-1 up-regulation of an individual.**
(XLS)

Table S11 **Output of the modeling system.**
(XLS)

Table S12 **Significantly de-regulated miRNAs (p-value <0.05) which are also listed in the PhenomiR database.**
(XLS)

Methods S1 **Supplementary methods.**
(DOCX)

Data S1 **SBML file of the Signaling Cancer Model.**
(XML)

Data S2 **Validation of the Illumina sequencing data.**
(DOCX)
