# Supplementary material for: High-Throughput miRNA and mRNA Sequencing of Paired Colorectal Normal, Tumor and Metastasis Tissues and Bioinformatic Modeling of miRNA-1 Therapeutic Applications
Source: PLoS One. 2013 Jul 2;8(7):e67461. doi: 10.1371/journal.pone.0067461 (PMC3707605; doi:10.1371/journal.pone.0067461)
Supplement: Figure S1 — Changes in miRNA expression between normal, tumor and metastasis tissues. (PPT) [file pone.0067461.s001.ppt]

## Slide 1
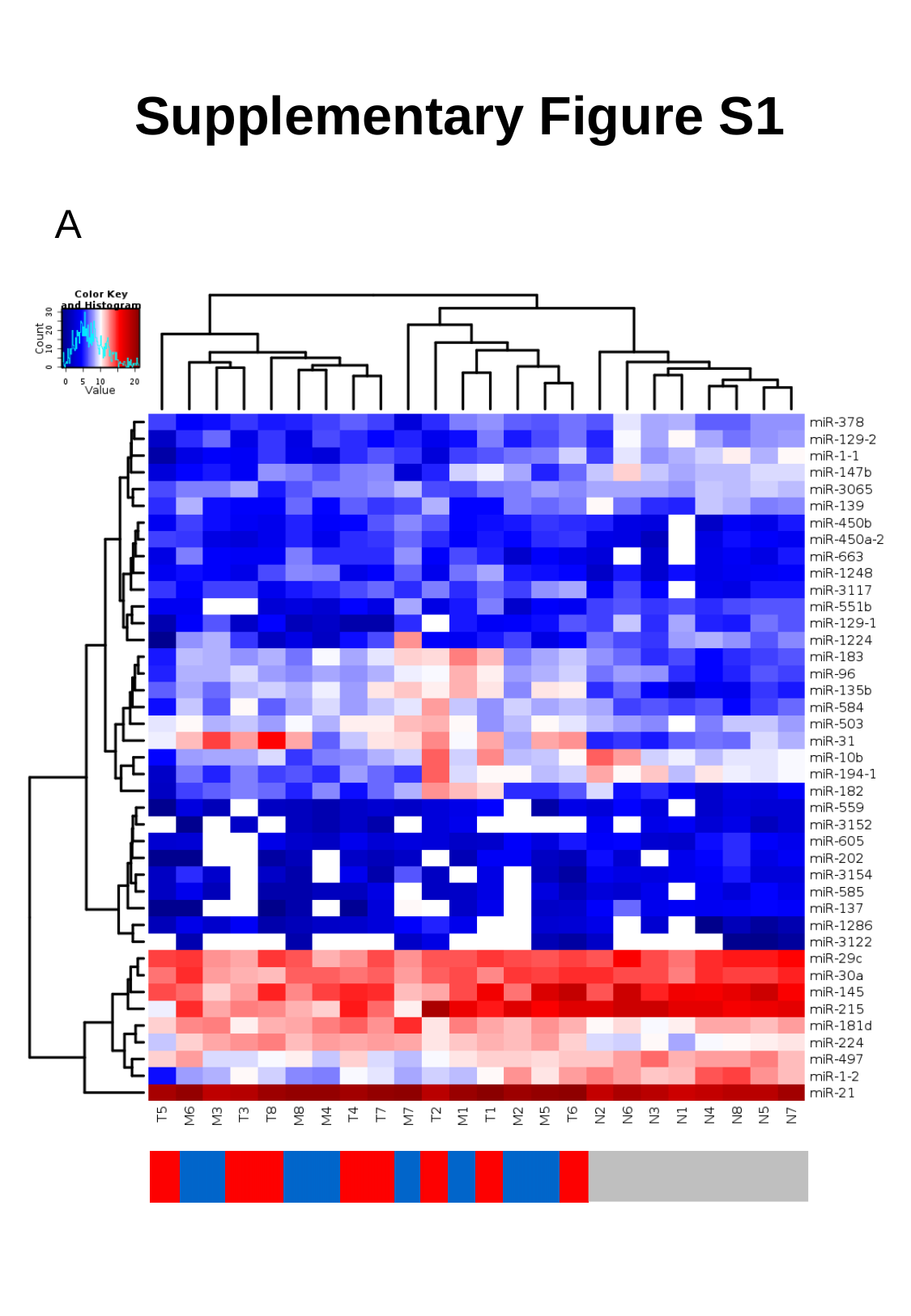

Supplementary Figure S1
A

## Slide 2
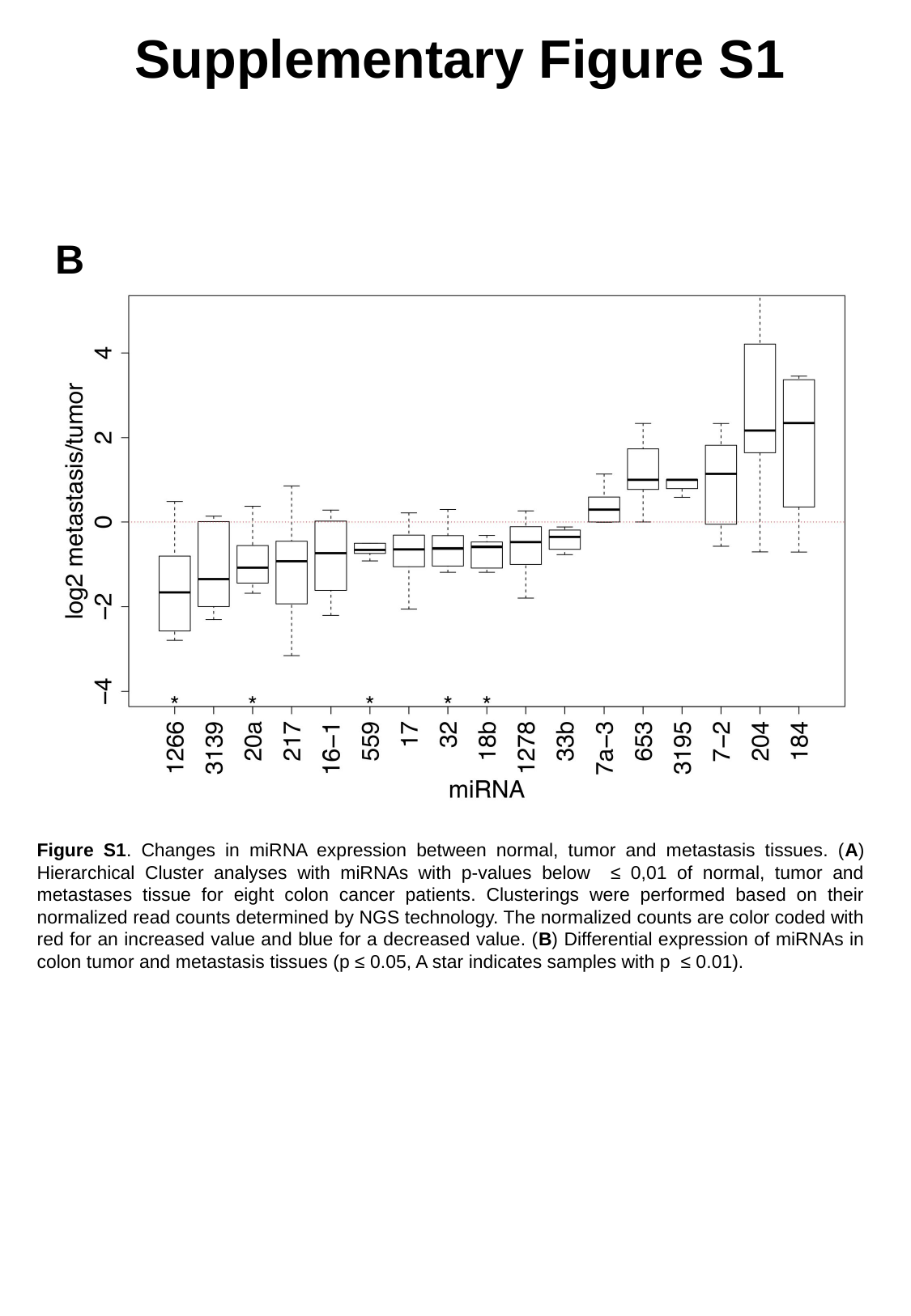

Supplementary Figure S1
B
Figure S1. Changes in miRNA expression between normal, tumor and metastasis tissues. (A) Hierarchical Cluster analyses with miRNAs with p-values below ≤ 0,01 of normal, tumor and metastases tissue for eight colon cancer patients. Clusterings were performed based on their normalized read counts determined by NGS technology. The normalized counts are color coded with red for an increased value and blue for a decreased value. (B) Differential expression of miRNAs in colon tumor and metastasis tissues (p ≤ 0.05, A star indicates samples with p ≤ 0.01).
