# Supplementary material for: High-Throughput miRNA and mRNA Sequencing of Paired Colorectal Normal, Tumor and Metastasis Tissues and Bioinformatic Modeling of miRNA-1 Therapeutic Applications
Source: PLoS One. 2013 Jul 2;8(7):e67461. doi: 10.1371/journal.pone.0067461 (PMC3707605; doi:10.1371/journal.pone.0067461)
Supplement: Figure S2 — Modeling of the miRNA down- and upregulation in metastasis tissues of patient 3,4,5 and 7. (PPT) [file pone.0067461.s002.ppt]

## Slide 1
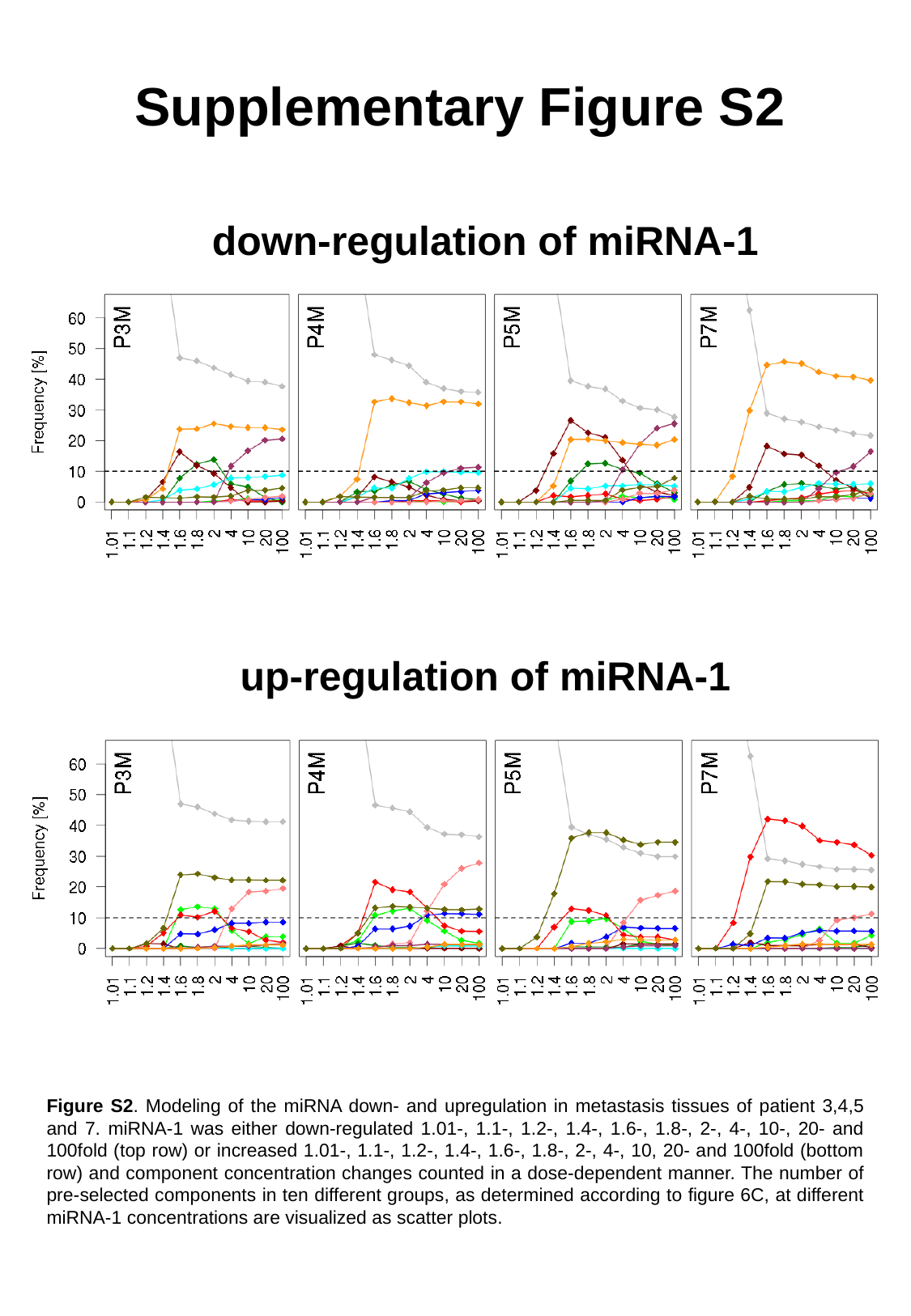

Supplementary Figure S2
down-regulation of miRNA-1
up-regulation of miRNA-1
Figure S2. Modeling of the miRNA down- and upregulation in metastasis tissues of patient 3,4,5 and 7. miRNA-1 was either down-regulated 1.01-, 1.1-, 1.2-, 1.4-, 1.6-, 1.8-, 2-, 4-, 10-, 20- and 100fold (top row) or increased 1.01-, 1.1-, 1.2-, 1.4-, 1.6-, 1.8-, 2-, 4-, 10, 20- and 100fold (bottom row) and component concentration changes counted in a dose-dependent manner. The number of pre-selected components in ten different groups, as determined according to figure 6C, at different miRNA-1 concentrations are visualized as scatter plots.
