# Supplementary material for: High-Throughput miRNA and mRNA Sequencing of Paired Colorectal Normal, Tumor and Metastasis Tissues and Bioinformatic Modeling of miRNA-1 Therapeutic Applications
Source: PLoS One. 2013 Jul 2;8(7):e67461. doi: 10.1371/journal.pone.0067461 (PMC3707605; doi:10.1371/journal.pone.0067461)
Supplement: Figure S3 — Marker combinations for the discrimination of normal/tumor, normal/metastasis, normal/tumor and metastasis and tumor/metastasis tissues. (PPT) [file pone.0067461.s003.ppt]

## Slide 1
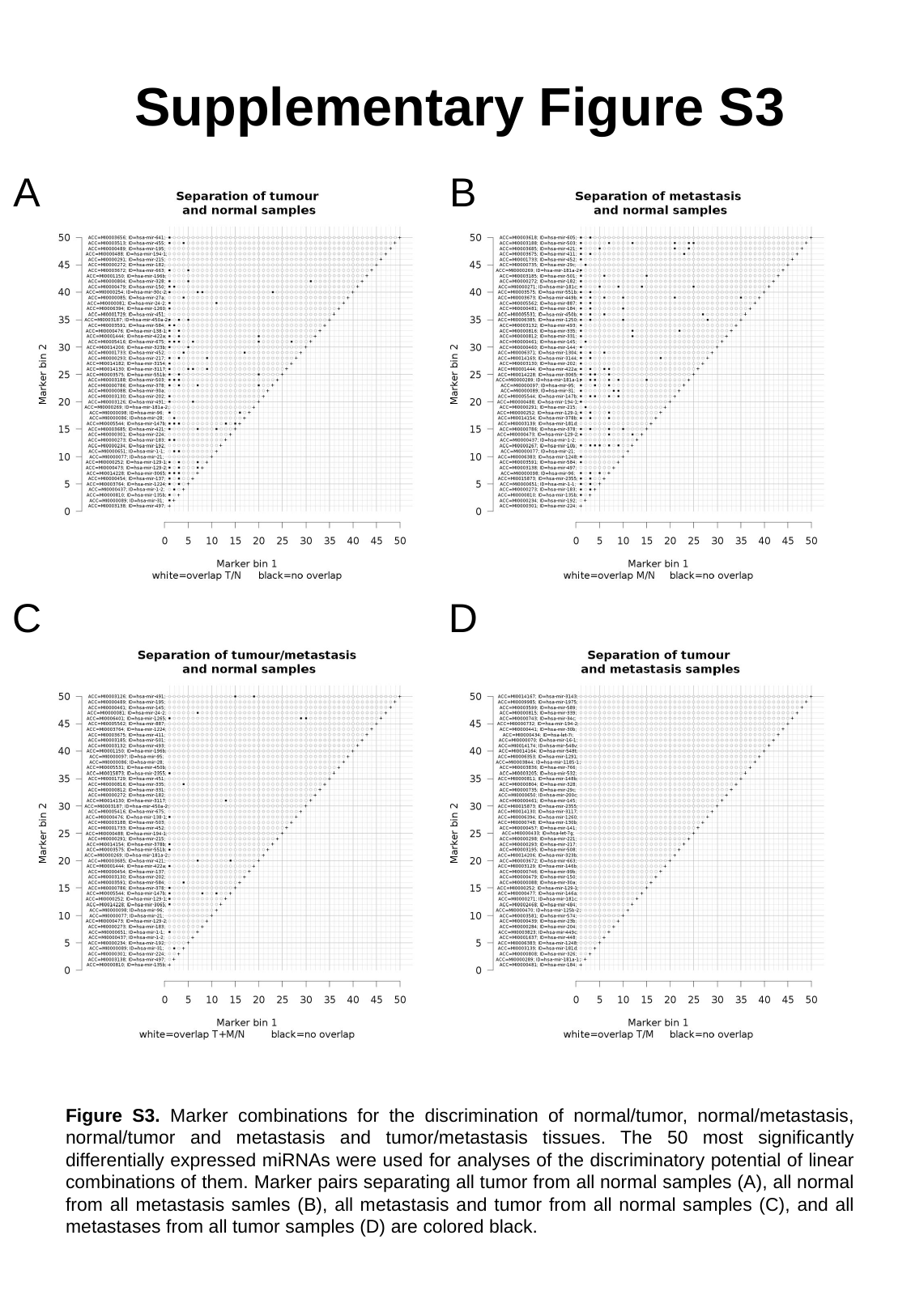

Supplementary Figure S3
A
B
D
C
Figure S3. Marker combinations for the discrimination of normal/tumor, normal/metastasis, normal/tumor and metastasis and tumor/metastasis tissues. The 50 most significantly differentially expressed miRNAs were used for analyses of the discriminatory potential of linear combinations of them. Marker pairs separating all tumor from all normal samples (A), all normal from all metastasis samles (B), all metastasis and tumor from all normal samples (C), and all metastases from all tumor samples (D) are colored black.
