# Supplementary material for: High-Throughput miRNA and mRNA Sequencing of Paired Colorectal Normal, Tumor and Metastasis Tissues and Bioinformatic Modeling of miRNA-1 Therapeutic Applications
Source: PLoS One. 2013 Jul 2;8(7):e67461. doi: 10.1371/journal.pone.0067461 (PMC3707605; doi:10.1371/journal.pone.0067461)
Supplement: Methods S1 — Supplementary methods. (DOCX) [file pone.0067461.s016.docx]

Supplementary Methods S1

Modeling of miRNA-1 function and therapeutic effects

The model comprises more than 4,000 components representing functional interactions of about 500 genes. The model defines synthesis and decay reactions for each protein where the protein synthesis rate is defined proportional to experimental expression data. The signal transduction network takes into account catalytic modifications (e.g. protein phosphorylation), protein-protein interactions and translocations.

In detail we defined three states as normal N_0_, tumor T_0_ and metastasis M_0_ (**Figure 4A**). The default values of all other objects in the model were zero. In this way, we 'copied' the genetic information of gene expression data from an individual sample into our model system.

In order to investigate the effect of miRNA-1 overexpression and repression on the biological system of an individual, we screened our cancer model for target genes of miRNA-1. According to the miRWalk database (version March 15^th^ 2011, ([Dweep et al., 2011](#_ENREF_7))) 843 genes are known as validated targets of miRNA-1, of which 59 are integrated in our cancer model (**Supplementary Table S6**). To study the quantitative effect of different miRNA-1 levels, we simulated different levels of the miRNA target genes and extracted the expression levels of all 1645 components contained within the model (**Supplementary Table S7, S8**).

The states with artificially altered miRNA-1 concentrations were defined as T_1_ to T_y_ in the case of miRNA-1 down-regulation and T_1_ to T_z_ in the case of miRNA-1 up-regulation.

Outputs of the simulations are steady state levels of expression which we used to calculate the effect of different miRNA-1 levels in terms of changed expression. The changed expression levels for all genes in the model were then evaluated according to whether the effect was desired or adverse.

In detail, differential expression was calculated as comparison between tumor (T_0_) and normal state (N_0_) as well as between treated (T_1_ to T_y_ or T_1_ to T_z_) and normal (N_0_) states.

For downstream analyses only those model components were considered as being affected by these different folds of miRNA-1 down-regulation during simulation, when they were more than 1.2-fold up- or 0.8-fold down-regulated with comparison to the control state.

**Tissue samples and cell lines**

The primary colon carcinoma tissue and matched normal colon epithelium as well as liver metastases tissue used for the next generation sequencing (NGS) experiments were obtained from patients diagnosed with CRC and undergoing surgical resection (**Table 1**). The samples were snap frozen in liquid nitrogen immediately after surgery and stored in liquid nitrogen. The study has been approved by the Research Ethics Committee of the Medical University of Graz. In total we analyzed 8 human colorectal cancer patients with TNM state T4N1M1. The sample set encompassed four male and four female patients between the age of 44 and 80 years with proximal and distal localizations of the tumor. The set also included microsatellite stable and instable tumors.

**RNA isolation from human cell lines**

RNA was extracted with the Trizol (Invitrogen) reagent according to the manufacture’s protocol. In brief, cells were grown on a 12-well plate and harvested by addition of 150µl Trizol. The cells were scraped off 30µl chloroform were added to separate phases. Samples were mixed vigorously and incubated for 2-3 minutes on ice before centrifugation at 4°C, 12000xg for 15 minutes. The upper phase was transferred to a new collection tube and 1ul of GenElute-LPA as well as 150µl of isopropyl alcohol were added. The RNA was precipitated at -80°C and pelleted by centrifugation at 12000xg for 10 minutes. The pellets were washed in 75% ethanol, air dried and diluted in 87.5µl RNase-free water. To eliminate the remaining DNA from the RNA solution a DNase digestion step was performed using the RNase-free DNase set from Qiagen according to the manufacture’s protocol. Therefore 10µl of the RDD buffer and 2.5µl DNase I were added to a final volume of 100µl of the solution. The sample was incubated for 10 minutes at room temperature and afterwards cleaned. For cleaning procedure the 100µl solution was transferred to a Phase lock tube (heavy) and phenol/chloroform/isopropyl alcohol 25/24/1 was added to the sample, mixed vigorously and incubated for 5 minutes at room temperature. To separate the organic phase from the aqueous RNA containing phase the samples were centrifuged for 5 minutes at 11000 rpm. The upper phase was transferred to a new collection tube. For ethanol precipitation 1µl GenElute, 0.2 volume 10M ammonium acetate and 2 volumes 100% ethanol were added to the sample and mixed vigorously before they were incubated at -80°C for approximately 30 minutes to precipitate the RNA. After incubation the samples were centrifuged for 15 minutes at full speed at 4°C to pellet the RNA. The supernatant was discarded and the pellet was washed in 500µl 75% ethanol and air dried. The RNA pellet was finally resuspended in 30µl RNase-free water. The integrity was assessed using the Agilent BioAnalyzer 2100 technology.

**Library preparation and next generation sequencing of RNAs and small RNAs.**

SmallRNA preparation from human colon tissues and cell lines for Illumina Genome Analyzer GAII sequencing included the following steps: smallRNA isolation, cDNA library preparation, sequencing and was performed by Illumina’s DGE smallRNA sample prep kit following the manufacture’s instructions. In short, 10µg total RNA was size-fractionated on a denaturing 15% TBE-urea polyamide gel and smallRNA fragments of 18-30 bases in length were purified from the gel Therefore the gel was centrifuged at 14000 rpm for 2 minutes through the punctured bottom of the 0.5 ml tube into the 2 ml tube to crush the piece of gel. To the gel debris 300µl of SRA 0.3M NaCl were added and the RNA was eluted by rotating the tube gently at room temperature for 4 h. The eluate and the gel were transferred to the top of a Spin X cellulose acetate filter and centrifuged for 2 minutes at 14000 rpm to separate the gel from the eluate. To precipitate the RNA 1µl glycogen and 750µl of room temperature 100% ethanol were added and incubated at -80°C for 30 minutes. Then the sample was centrifuged immediately at 14000 rpm for 25 minutes at 4°C. The supernatant was removed and discarded before the pellet was washed in 750µl of 75% room temperature ethanol. After letting the pellet dry it was resuspended in RNase-free water. To generate cDNAs the Illumina 3’ adapter (5'-P-UCGUAUGCCGUCUUCUGCUUGUidT-3’) and 5’ RNA adapter (5'-GUUCAGAGUUCUACAGUCCGACGAUC-3’) were ligated to the size selected RNA molecules to enable reverse transcription, an amplification step as well as the sequencing procedure. To remove unligated adapters, the ligation products of the desired size range of 70-90 bases in length were purified from a 10% TBE-urea polyacrylamide gel as described before. The precipitated RNA was converted to single-stranded cDNA using Superscript II revese transcriptase (Invitrogen) and Illumina’s smallRNA RT-Primer (sequence). After a PCR-amplification step with15 cycles using Phusion DNA Polymerase and Illumina’s small RNA primer set (Small RNA PCR Primer 1: 5'-CAAGCAGAAGACGGCATACGA-3’; Small RNA PCR Primer 2: 5' AATGATACGGCGACCACCGACAGGTTCAGAGTTCTACAGTCCGA-3’). The amplification products were separated on a 6% TBE polyacrylamide gel. The corresponding gel fragment of approximately 92 bases in length was excised. The purified DNA was quantified and diluted to 10nM for cluster generation and sequencing on an Illumina Genome Analyzer GAII.

As reference gene RNU44 was used. Other reports suggest miRNA-16, miRNA-26 or miRNA-345 as references. We find that each of these small RNAs – including RNU44 - performs equally well on our data set (Figure). Normalization results in a constant down-regulation of miRNA-1 with comparable variability irrespective of the reference gene used (lane d and e) – or even without reference gene normalization (lane c).


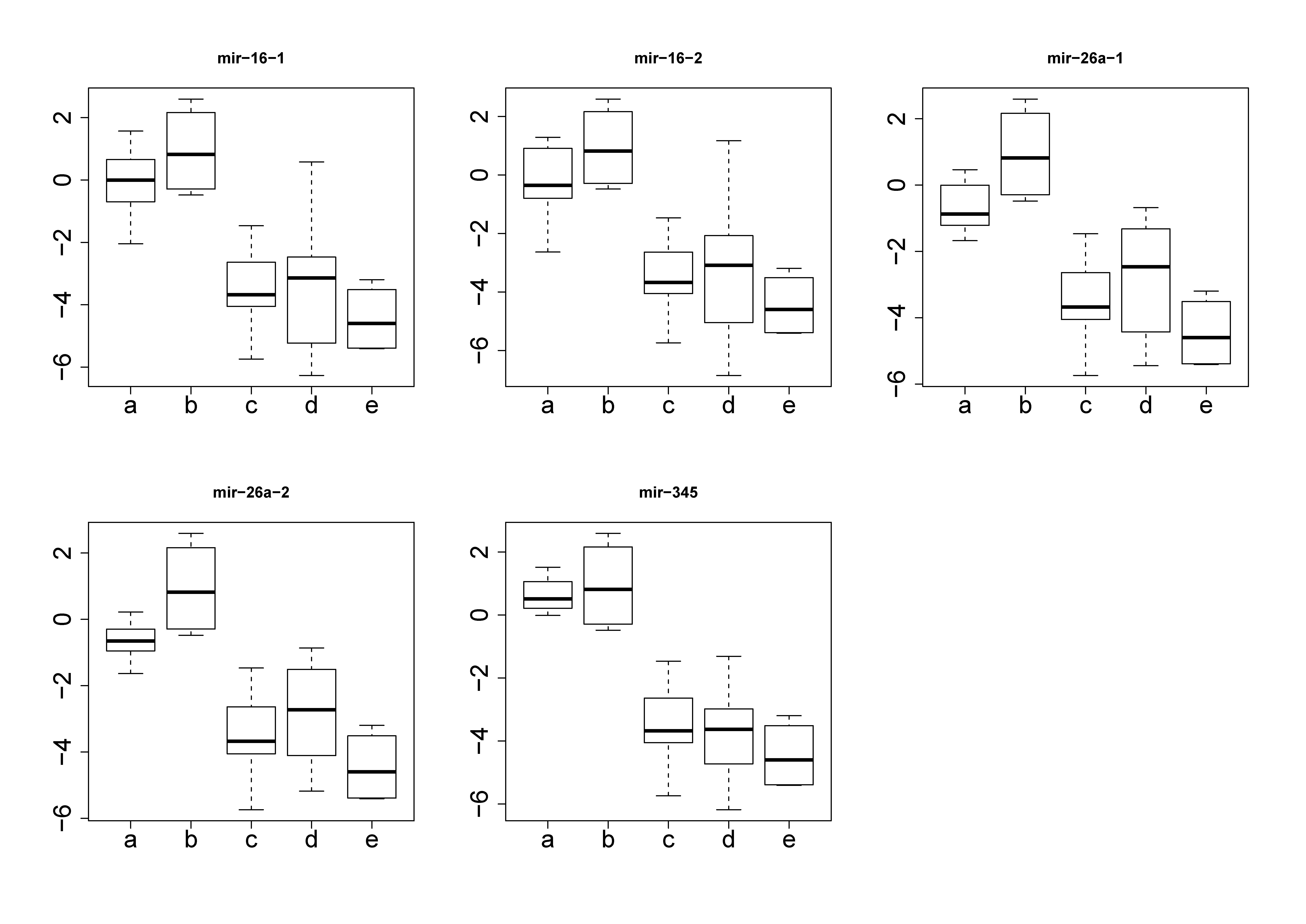


**Figure** Expression of candidate reference gene small RNAs, miRNA-1 and different normalization strategies. Depicted are log2 (read) – ratios of tumor vs benign tissues. Lane a) ratio of the proposed control miRNA, b) ratio of RNU44, c) ratio of miRNA-1, d) ratio of miRNA-1 normalized to the proposed miRNA, e) ratio of miRNA-1 normalized to RNU44.

**DNA sequencing library preparation for Solexa**

For RNA sequencing transcriptome isolation was performed applying RiboMinus™ Human/Mouse Transcriptome Isolation Kit. Therefore human rRNA sequence-specific 5’-biotinylated oligonucleotide probes and streptavidin coated magnetic beads were used. As input amount 2µg of total RNA in a 20µl volume diluted in RNase-free water were used. To the RNA sample 300µl Hybridization Buffer B5 8µl of the RiboMinus probe were added and incubated for 5 minutes at 75°C. Then the sample was cooled down slowly to 37°C over the time of 30 minutes to hybridize the rRNA to the 5’-biotin labeled probe. While the sample was cooling down the streptavidin coated RiboMinus™ Magnetic Beads were prepared. Therefore 500µl of the beads per sample were pipetted into a 1.5 ml microcentrifuge tube and placed for 1 minute on a magnetic stand. During this procedure the beads settled to the bottom of the tube. The supernatant was aspirated and discarded. For washing the beads 500µl of RNase-free water was pipetted to the beads and resuspended by inverting the and gently tapping the tube. The tube with the bead suspension was placed on a magnetic stand for 1 minute and the supernatant was removed. The water cleaning step was repeated and followed by re-suspension step in 500µl Hybridization Buffer B5. The tube was placed on a magnetic stand for 1 minute and the supernatant removed. Until hybridization the bead pellet was diluted in 200µl Hybridization Buffer B5 and incubated at 37°C. The hybridization sample was collected by centrifugation briefly and the 328µl hybridization sample was added to the prepared beads and mixed well. The solution was incubated for 15 minutes at 37°C. During incubation the rRNA/5’-biotin labeled probe and bead complex was gently mixed occasionally. The tube was placed on a magnetic stand for 1 minute and the supernatant (~528µl) was transferred to a new 2 ml collection tube. For precipitating the RNA from this solution 1µl GenElute LPA 50µl of 10M NH_4_Ac and 600µl Isopropanol were added and incubated for 30 minutes at -80°C. Afterwards it was centrifuged for 15 minutes at 4°C at full speed. The pellet was washed in 70% ethanol and air dried. To resolve the pellet 10µl RNase-free water were added. After elimination of abundant large ribosomal RNA molecules from total RNA, cDNA synthesis was performed. Therefore we applied the 10µl RNA solution isolated by RiboMinus™ Human/Mouse Transcriptome Isolation Kit and added 4.7µl random hexamer primer of a 50ng/µl stock solution 1.25µl dNTPS (10mM each). The solution was heated to 65°C for 5 minutes and quick chilled on ice. After cooling down the sample 2.5µl DDT (0.1M) 5µl of 5X first strand buffer and 0.5µl RNaseOut were added and incubated for 2 minutes at 25°C before 1µl SuperScript was added. The sample was incubated for additional 10 minutes at 25°C and then heated up to 42°C for 50 minutes to initiate the activity of the reverse transcriptase before the sample was heated up to 70°C for 15 minutes and afterwards quick chilled on ice. To the 25µl of the first strand reaction 85µl water 30µl second strand buffer 3µl dNTPs (10mM each) 2µl E. coli DNA Ligase (10U/µl) 4µl E. coli DNA Polymerase I (10U/µl) and 2U E. coli RNaseH were added. The solution was incubated at 16°C for 2h and cooled on ice. For final sample purification the 150µl of the synthesized dsDNA were transferred to a Phase Lock tube (light) and 150µl Phenol/Chloroform/Isopropyl alcohol 25:24:1 were added and mixed vigorously and incubated for 5 minutes at room temperature before centrifuged for 5 minutes at 11000 rpm. The upper dsDNA containing phase was transferred to a new collection tube and 1µl GenElute LPA 0.2 volume 10M ammonium acetate and 100% ethanol were added. The solution was incubated at -80°C for 30 minutes and afterwards centrifuged. The pellet was washed with 500µl 70% ethanol and air dried. The pellet was finally resuspended in 10µl water and the concentration was measured using NanoDrop technology. The dsDNA was used to prepare a sequencing cDNA library which was fragmented to a general size of less than 800bp.

Therefore an ultrasonic bath (Sonifier cell disruptor B-30) was used. Up to 5 µg of the DNA were used as input amount. End repair reaction was performed and an ‘A’ base was added to the 3'-end of the blunt phosphorylated DNA fragments before the adapters were ligated for hybridization to a flow cell. A final sample purification and size selection of the generated library fragments was performed over gel electrophoresis and the obtained library fragments were applied to the cluster generation platform before sequencing on Illumina.

**Real-time quantification of microRNAs using stem-loop real-time PCR**

For real-time quantification of mature microRNAs we used TaqMan® MicroRNA Assays (Applied Biosystems) and performed a two-step RT-PCR according to the manufacturer’s protocol: In the reverse transcription reaction, single-stranded cDNA was synthesized from 25ng total RNA using 50nM looped RT primer (PN 4427975), 1X Reverse Transcription buffer, 0,25mM each of dNTPs, 0,25U/µl RNase Inhibitor and 3,33U/µl MultiScribe™ Reverse Transcription per 15µl reaction (TaqMan® MicroRNA Reverse Transcription Kit, Applied Biosystems PN 4366596). The reaction was incubated in a thermal cycler (Applied Biosystems 9700 Thermocycler) for 30 min at 16°C, 30 min at 42°C, followed by 5 min incubation at 85°C to inactivate the Multiscribe Reverse Transcriptase and then hold at 4°C. The cDNA was diluted 10-fold before preparing for PCR reaction.

For the quantification of the RT-products TaqMan® minor groove binding (MGB) probes were used in a 10µl PCR reaction, containing 1µl of the 10-fold diluted cDNA, 1X TaqMan® MicroRNA Assay (PCR Primer/Probe/dNTP mix) and 1X TaqMan® Universal PCR master mix. The real-time PCR was performed in a 384-well plate using standard protocols on an Applied Biosystems 7900HT Fast Real-Time PCR System, including 10 min incubation at 95°C to activate the AmpliTaq® Gold enzyme, followed by 40 amplification cycles of 95°C for 15 s and 60°C for 1 min. All reactions were typically run in triplicates. The relative quantification of expression was calculated by the comparative "delta CT" method using RNU44 as an internal control. The fold change is described as 2^-delta delta Ct^ value for real-time experiments ([Schmittgen and Livak, 2008](#_ENREF_23)). The comparison of normal and tumor or metastasis tissues was calculated as log2-ratios. Default threshold settings were applied to determine the Ct value, which is defined as the fractional cycle number at which the fluorescence passes the fixed threshold ([Schmittgen and Livak, 2008](#_ENREF_23)).
