# Supplementary material for: High-Throughput miRNA and mRNA Sequencing of Paired Colorectal Normal, Tumor and Metastasis Tissues and Bioinformatic Modeling of miRNA-1 Therapeutic Applications
Source: PLoS One. 2013 Jul 2;8(7):e67461. doi: 10.1371/journal.pone.0067461 (PMC3707605; doi:10.1371/journal.pone.0067461)
Supplement: Data S2 — Validation of the Illumina sequencing data. (DOCX) [file pone.0067461.s018.docx]

**Supplementary Data S2. Validation of the Illumina sequencing data.**

We probed the reliability of o our high-throughput sequencing approach using technical replicates of the smallRNA fractions of two colorectal cancer cell lines (SW480 and SW620) and achieved Pearson correlation coefficients of 0.97 for SW480 and 0.86 for SW620 (Supplementary data Fig. S1). We furthermore validated the sequencing data with TaqMan assays for four up-regulated (miRNA-31, miRNA-135b, miRNA-183, miRNA-96) and six down-regulated microRNAs (miRNA-1, miRNA-145, miRNA-133a, miRNA-133b, miRNA-150, miRNA-375) in up to sixteen patients and achieved a Pearson’s correlation coefficient of 0.89 (Supplementary data Fig S2). To validate the miRNA expression analysis strategy we compared sequencing data to TaqMan data for 10 different miRNAs in up to 8 patients: Normalized log2 ratios from both datasets were plotted against each other and a linear model (y = mx + b) was fitted for two comparisons: tumor vs benign and metastasis vs benign. We calculated the Pearson product-moment correlation for both comparisons to demonstrate the agreement of both datasets. Hierarchical clustering were performed with the heatmap.2-function included in the R package ‘gplots’ using default parameters.

**Supplementary Data Figure S1** Correlation of NGS and real-time PCR analyses of miRNAs. Depicted are the log2 ratios for 10 different miRNAs in up to 8 patients as determined by both technologies for the comparisons tumor (red) and metastasis (blue) versus normal. The regression analyses resulted in Pearson correlation coefficients of 0.89 for both comparisons. The identity line (y=x) is depicted in green.

**Supplementary data Figure S2**. Validation of microRNA expression pattern generated by NGS technology, using TaqMan assays for mature miRNAs. (A) Three CRC patients were analyzed on a set of 10 microRNAs (miRNA-31, miRNA-135b, miRNA-183, miRNA-96, determined as up-regulated and miRNA-1, miRNA-145, miRNA-133a, miRNA-133b, miRNA-150, miRNA-375, determined as down-regulated). The expression values were calculated using the delta delta Ct method, using RNU44 as an internal control gene. The log2ratio of tumor versus normal (top) and metastasis versus normal (bottom) was plotted for all three patients (P1-P3). (B) For a set of 4 microRNAs (miRNA-1, miRNA-145 predicted as down-regulated and miRNA-135b, miRNA-31 predicted as up-regulated) we tested the expression in tumor tissues on a larger sample set of 16 CRC patients (top). For the metastases we checked the expression levels of 8 sequenced CRC patients (bottom). The log2ratio of each patient was calculated using the delta delta Ct method with normalizations against the small nuclear RNA RNU44.
